# Generalized X-ray and neutron crystallographic analysis: more accurate and complete structures for biological macromolecules

**DOI:** 10.1107/S0907444909011548

**Published:** 2009-05-15

**Authors:** Paul D. Adams, Marat Mustyakimov, Pavel V. Afonine, Paul Langan

**Affiliations:** a Lawrence Berkeley National Laboratory, CA 94720, USA; b Department of Bioengineering, UC Berkeley, CA 94720, USA; c Los Alamos National Laboratory, NM 87545, USA

**Keywords:** joint X-ray and neutron crystallography, structure refinement

## Abstract

X-ray and neutron crystallographic techniques provide complementary information on the structure and function of biological macromolecules. X-ray and neutron (XN) crystallo­graphic data have been combined in a joint structure-refinement procedure that has been developed using recent advances in modern computational methodologies, including cross-validated maximum-likelihood target functions with gradient-based optimization and simulated annealing. The XN approach for complete (including hydrogen) macromolecular structure analysis provides more accurate and complete structures, as demonstrated for diisopropyl fluorophosphatase, photoactive yellow protein and human aldose reductase. Furthermore, this method has several practical advantages, including the easier determination of the orientation of water molecules, hydroxyl groups and some amino-acid side chains.

## Abstract 2

X-ray and neutron crystallographic data have been combined in a joint structure-refinement procedure that has been developed using recent advances in modern computational methodologies, including cross-validated maximum-likelihood target functions with gradient-based optimization and simulated annealing.

## 1. Introduction   

Although X-ray and neutron crystallography are both long-established methods, the greater brightness of X-ray sources makes the former the method of choice for determining the structures of biological macromolecules. However, the dependence of X-ray scattering on atomic electron number makes H atoms difficult to locate in experimental electron-density maps. Neutron crystallography is a powerful technique for locating hydrogen and can readily provide information on the protonation states of amino-acid residues and ligands, the identity of solvent molecules and the nature of bonds involving hydrogen (Niimura & Bau, 2008 ▶). Neutron crystallo­graphy can also be used to identify H atoms that are exchanged with deuterium and the extent of this replacement, thus providing a tool for identifying isotopically labeled structural features, for studying solvent accessibility and macromolecular dynamics and for identifying minimal protein-folding domains (Bennett *et al.*, 2008 ▶). The uniqueness of this type of information and its complementarity to the information provided by X-ray diffraction has given neutrons a small but important role in biology, notably in determining the details of the catalytic mechanisms, functions, drug binding and other properties of biological macromolecules (Blakeley, Langan *et al.*, 2008 ▶).

Recently, there has been a great increase in the application of neutrons in biology, mainly owing to improvements in instrumentation and data-collection and sample-preparation methods (Blakeley, Langan *et al.*, 2008 ▶). Although there is still a current need for large crystals, or full deuteration methods when crystals are small, the commissioning of several new and more powerful neutron sources promises routine data collection within a few years from crystals that are of similar size to those used for X-ray crystallography (Teixeira *et al.*, 2008 ▶). This increasing data-collection throughput, together with the increasing size and complexity of the systems now being studied, is uncovering obstacles and bottlenecks in structure determination. Neutron structure refinement is typically carried out separately from, and subsequent to, X-ray structure determination, but can be complicated for a number of reasons. At the same time, the increasing availability of both X-ray and neutron crystallographic data provides an opportunity to develop a generalized method for structure analysis that exploits the complementarity of these data in order to provide a more accurate and complete structure.

In response to these problems and opportunities, we have developed joint X-ray and neutron structure refinement, which was originally applied to small molecules (Coppens *et al.*, 1981 ▶), and combined it with recent advances in modern structure-refinement methodology to provide a new generalized method for complete (including hydrogen) structure refinement. This method, which we designate XN, involves the collection of both neutron and X-ray data sets at the same temperature from crystals grown under similar conditions. We demonstrate that when the XN method is applied to several biological macromolecules involved in a range of different biological processes the XN structures are more complete and more accurate than the structures determined using either the X-ray or neutron data independently.

## 2. Methods   

### 2.1. Rationale for XN refinement   

**Figure 1:**
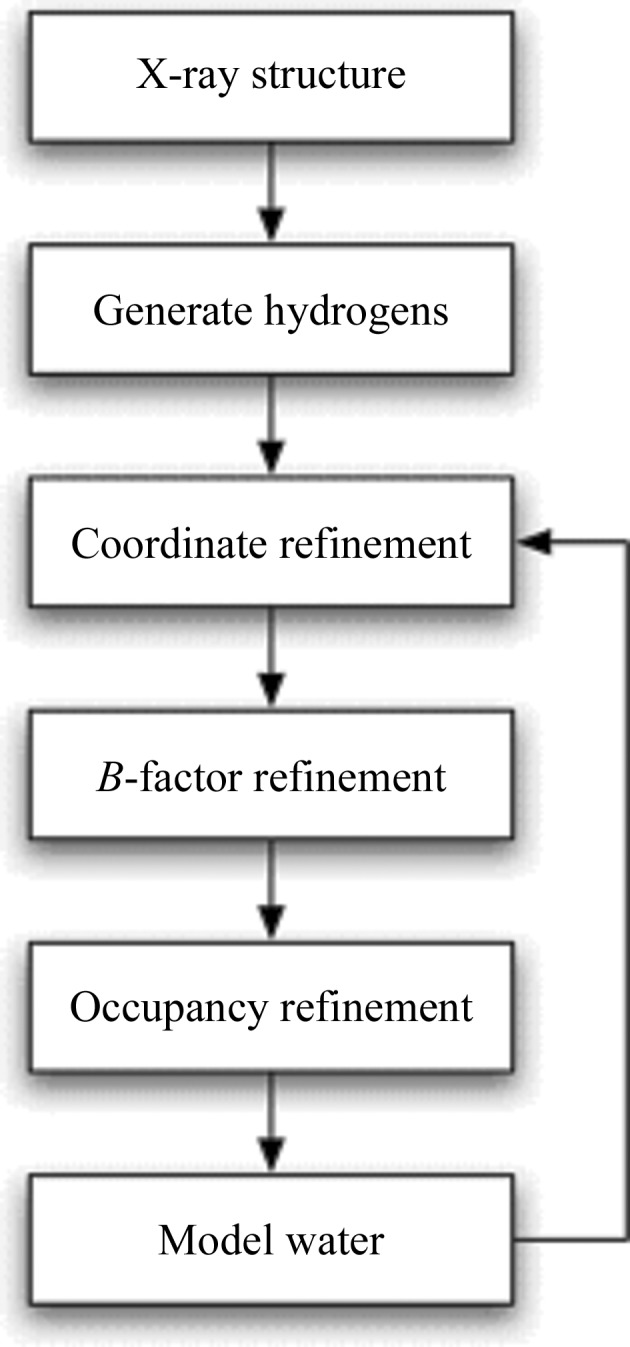
General strategy for neutron crystallographic refinement of biological macromolecules. The X-ray model serves as a starting point; H atoms are subsequently added and the model is optimized to obtain the best agreement with the observed neutron data. The initial positions of some H atoms can be predicted from the known geometries of certain functional groups. For others, particularly those in solvent molecules, initial positions can only be determined from inspecting the neutron scattering density map. The proportions of hydrogen and deuterium at labile sites must also be refined in cases where the crystal has been grown or soaked in D_2_O to enhance its neutron scattering properties. D atoms (neutron scattering length of 6.67 × 10^−15^ m) appear as strong positive peaks in neutron scattering density maps, thereby revealing the location of isotopically exchanged H atoms and enhancing the scattering power of water molecules, whilst H atoms themselves (neutron scattering length of −3.7 × 10^−15^ m) appear as negative troughs. For amino-acid side chains that can be ionized, the neutron scattering density maps must be inspected to determine whether or not a H or D atom is present and in what position.

Neutron crystallography is used to determine the location of H atoms and their level of (hydrogen/deuterium) exchangeability or accessibility using the general strategy outlined in Fig. 1 ▶, once the positions of the heavier atoms have been determined using X-ray crystallography. Several limitations are associated with this process. Adding hydrogen can greatly increase the number of parameters to be refined. The number of neutron data is often smaller than the number of X-ray data because of the relatively weak flux of available neutron beams. These two factors reduce the data-to-parameter ratio from its value in typical X-ray studies, increasing the danger of overfitting and decreasing the accuracy of the optimized neutron model. In addition, at moderate resolutions (*d* > 2 Å), the negative scattering length of an H atom (−3.7 × 10^−15^ m) will tend to cancel out the positive scattering length of a covalently bound heavier atom (6.65 × 10^−15^ m for carbon; 5.8 × 10^−15^ m for oxygen; 9.36 × 10^−15^ m for nitrogen). Lysine, for example, has a strong neutron scattering density associated with its isotopically exchanged side-chain terminal amine group (the neutron scattering length of deuterium is 6.67 × 10^−15^ m) but little residual scattering density for the non-exchanged aliphatic methylene groups. The chain will therefore be ill-defined in a neutron refinement.

These two limitations can be overcome by using the approach, first applied to proteins by Wlodawer & Hendrickson (1981 ▶, 1982 ▶), of using both X-ray and neutron data together in a joint (XN) least-squares refinement procedure. The increase in the number of combined data improves the data-to-parameter ratio. The complementarity between X-ray and neutron scattering densities ameliorates cancellation effects; for example, although having little residual neutron scattering density, the methylene groups of lysine scatter X-rays almost as well as the terminal amine group.

However, there are also limitations associated with joint XN least-squares refinement. H or D atoms can account for a large proportion of the scattering power of the unit cell. The neutron phases calculated from the X-ray starting model are therefore likely to have large errors. There is a danger of overfitting by introducing systematic errors because a least-squares target function does not take into account phase errors in the current model. In addition, some parts of the X-­ray model may not be accurately defined because of disorder. Those same parts may have relatively large neutron scattering densities and at the start of neutron refinement can be close to local minima in the least-squares target function.

These limitations can be overcome by taking advantage of recent developments in X-ray structure-refinement algorithms. Maximum-likelihood target functions have been developed that make better allowance for the effects of data quality, model errors and incompleteness (Pannu & Read, 1996 ▶; Bricogne & Irwin, 1996 ▶; Murshudov *et al.*, 1997 ▶; Adams *et al.*, 1997 ▶; Pannu *et al.*, 1998 ▶). Cross-validation, in the form of an *R*_free_, has been developed as an unbiased indicator of overfitting (Brünger, 1992 ▶). Cross-validation has also been introduced in calculating the terms in the maximum-likelihood target function (Urzhumtsev *et al.*, 1996 ▶). An automatic protocol for estimating the relative weight of X-ray and energy terms in the minimization has been adopted (Adams *et al.*, 1997 ▶). The use of molecular-dynamics simulated annealing can be used to reduce the dangers of local minima by increasing the radius of convergence (Brünger *et al.*, 1987 ▶; Adams *et al.*, 1997 ▶; Brunger & Adams, 2002 ▶).

We have incorporated a generalized XN refinement approach into two crystallographic software systems, *CNS* (Brünger *et al.*, 1998 ▶) and *PHENIX* (Adams *et al.*, 2002 ▶), in order to address the limitations discussed above. *CNS* was chosen as a test platform because it has a hierarchical structure: an HTML user interface, task-oriented user-input files, module files, a scripting language specialized for X-ray structure analysis (*CNS* language) and low-level source code. The *CNS* language is sufficiently powerful and flexible that new algorithms and methodologies can be implemented and tested without large changes to the low-level source code. *PHENIX* was chosen for long-term development because it has greater flexibility for accommodating further developments and provides a range of model parameterizations including, for example, anisotropic displacement parameters and TLS.

A detailed description of the implementation of this approach in *CNS* and *PHENIX* will be given elsewhere. Briefly, a version of *CNS* was adapted for XN refinement by making minor changes to the low-level source code and more extensive changes to the symbolic language. This adaptation is distributed free as a patch for *CNS*, called *nCNS*, from the website http://mnc.lanl.gov or by contacting the authors of this paper. A copyright (C-06,104) obtained by Los Alamos National Security, LLC allows *nCNS* to be distributed free under a GNU General Public License. *PHENIX*, a system being actively developed for automated X-ray structure refinement, was extended to include joint XN refinement. This built upon the fundamental algorithms implemented in the computational crystallography toolbox (CCTBX; Grosse-Kunstleve *et al.*, 2002 ▶). An existing set of external reference files for the definition of molecular topology and associated restraint, the *CCP*4 monomer library (Vagin *et al.*, 2004 ▶), was used to provide compatibility with a number of other crystallographic software packages. The new refinement tool, called *phenix.refine* (Afonine *et al.*, 2005 ▶), is made freely available to all nonprofit users and can be downloaded from http://www.phenix-online.org.

### 2.2. The generalized XN refinement method   

Although there are a number of differences in detail between the generalized XN method as implemented in *CNS* and *PHENIX*, the key common concept is the generalization of the target function to be minimized by the introduction of a neutron term
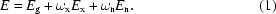
Here, *E* is a target function, *E*_g_ is a geometric or empirical energy term, *E*_x_ and *E*_n_ are the X-ray and neutron terms and ω_x_ and ω_n_ are weights. *E*_x_ and *E*_n_ can be least-squares, maximum-likelihood or phased maximum-likelihood functions. The generalized target function can therefore correspond to a number of possible combinations of least-squares, maximum-likelihood, X-­ray or neutron target functions. Different subsets of atoms can be used in calculating the X-ray and neutron terms; for example, H or D atoms can be excluded from the X-ray term while being incorporated in the neutron term. Estimates of ω_x_ and ω_n_ are calculated automatically using a comparison of root-mean-square gradients (Adams *et al.*, 1997 ▶). The role of ω_x_ and ω_n_ is to balance the relative contribution of different terms in the generalized target function.

Crystallographic *R*_work_ and *R*_free_ values for the X-ray (designated _x_ *R*_work_ and _x_ *R*_free_) and neutron (designated _n_ *R*_work_ and _n_ *R*_free_) terms are calculated to monitor the improvement of the fit to the crystallographic data during refinement and to detect overfitting. To calculate _x_ *R*_free_ and _n_ *R*_free_, a randomly selected ‘test’ set of reflections is set aside (typically 5–10%) and the remaining reflections in the ‘working’ set are used in the target function and in the calculation of _x_ *R*_work_ and _n_ *R*_work_. Despite being considered to be independent, neutron and X-­ray structure factors for the same reflection are correlated. In order to avoid bias in calculating _x_ *R*_free_ and _n_ *R*_free_, it is necessary to select a test set in such a way that those reflections used to calculate _x_ *R*_free_ and _n_ *R*_free_ are not used in the refinement of *E*_n_ or *E*_x_, respectively. Such a procedure has been implemented in both *CNS* and *PHENIX*.

If the automatic calculation of ω_x_ or ω_n_ is not optimal, the data may be overfitted, generating apparently good values for _x_ *R*_work_ and _n_ *R*_work_, but the geometry of the structure may be chemically unrealistic. We therefore monitor the gaps between _x_ *R*_work_ and _x_ *R*_free_ and between _n_ *R*_work_ and _n_ *R*_free_ and also the root-mean-square deviations of bond lengths and angles from their ideal values, designated *Δ*_bond_ and Δ_angle_, respectively. If necessary, one can run an automatic weight-optimization procedure in which an array of values is systematically tested to find the values that minimize _x_ *R*_free_ and _n_ *R*_free_. 

## 3. Results   

### 3.1. Validation of the generalized XN method   

**Figure 2:**
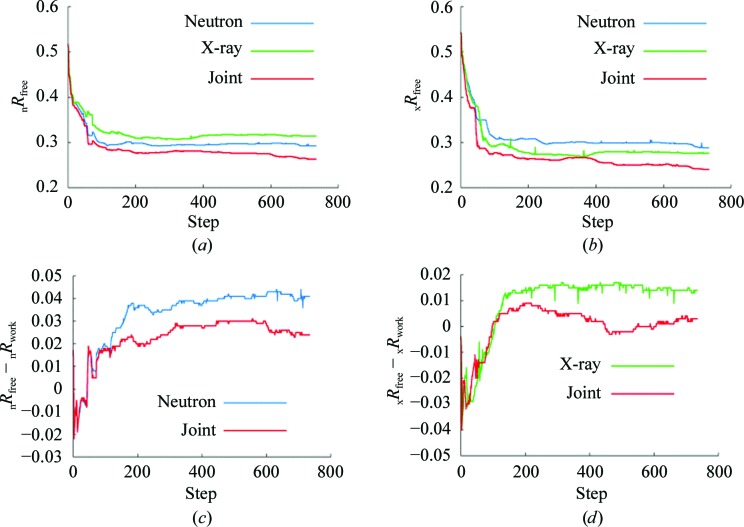
The behavior of crystallographic *R* factors during refinement of the W3Y rubredoxin mutant. The starting model has been distorted from the published model (r.m.s. coordinate error of 0.5 Å). The values of _n_ *R*_free_ (*a*) and _x_ *R*_free_ (*b*) are shown as a function of the number of cycles of refinement, where refinement was with either the X-ray data (green) or the neutron data (blue) alone or using both X-ray and neutron data (red). Also shown is the difference _n_ *R*_free_ − _n_ *R*_work_ (*c*) for refinement with either neutron data (blue) alone or using both X-ray and neutron data (red) and the difference _x_ *R*_free_ − _x_ *R*_work_ (*d*) for refinement with either X-ray data (green) alone or using both X-ray and neutron data (red). Some upward deviations in those plots are because new values of ω_x_ and ω_n_ were recalculated after each macrocycle.

Selected structures from the Protein Data Bank (Bernstein *et al.*, 1977 ▶; Berman *et al.*, 2002 ▶) for which both X-ray and neutron data sets were available were used to validate the generalized XN method, as implemented first in *CNS*. Models with systematic deviations from the original structure were generated by introducing random errors to the coordinates, atomic displacement parameters (ADP) and the levels of isotopic exchange of labile H atoms (with Gaussian distributions and linearly increasing r.m.s. displacements from the original values). These models were then used as starting points for maximum-likelihood optimization: 50 steps of coordinate refinement were followed by 20 steps of ADP refinement and then by 20 steps of labile H-atom occupancy refinement. A bulk-solvent correction was applied through­out. This macrocycle was repeated ten times. The final values of *R*_free_ for a W3Y rubredoxin mutant (Kurihara *et al.*, 2004 ▶; PDB codes 1iu5 and 1iu6) are shown in Table 1 ▶. These values are consistently smaller after joint XN refinement than after refinement using either the neutron or X-ray data on their own. The behavior of _x_ *R*_free_ and _n_ *R*_free_ during the progress of these refinements from one particular starting model (r.m.s. coordinate error of 0.5 Å) is shown in Figs. 2 ▶(*a*) and 2 ▶(*b*). A similar trend towards lower values for joint XN refinement compared with refinement with either only X-ray or neutron data as the refinement progresses was observed for all ten scrambled models. The differences between _x_ *R*_free_ and _x_ *R*_work_ and between _n_ *R*_free_ and _n_ *R*_work_ during the progress of the refinements represented in Figs. 2 ▶(*a*) and 2 ▶(*b*) are shown in Figs. 2 ▶(*c*) and 2 ▶(*d*). The differences are smaller for joint XN refinement than for refinement against neutron or X-ray data sets alone, indicating that joint XN refinement is less susceptible to overfitting of the data.

Encouraged by the results of the initial tests with *CNS*, we then implemented the joint XN refinement method in *PHENIX*. The validation process was slightly different, taking advantage of the availability of more sophisticated algorithms for automatic water building and constrained refinement of alternate conformations in *PHENIX*. Again, several structures from the PDB for which both X-ray and neutron data were available were used. All water molecules were removed, a random 1.5 Å shift was added to the coordinates and the values of the ADP were set to 15.0 Å^2^ for all initial models. Three batches of ten macrocycles of refinement were carried out for each model corresponding to joint XN refinement and refinement against either X-ray or neutron data alone. Each macrocycle consisted of automatic water building, refinement of individual coordinates and isotropic or anisotropic ADP and constrained refinement of occupancies for atoms in alternative conformations or exchangeable H/D sites. A bulk-solvent correction was applied throughout. These tests confirmed our initial results with *nCNS*: consistently lower or similar values were obtained with joint XN refinement. For the W3Y rubredoxin mutant, the *nCNS* validation tests of which are represented in Table 1 ▶, the final values of _x_ *R*_work_ and _x_ *R*_free_ for X-­ray refinement were 12.3 and 16.2, respectively, the final values of _n_ *R*_work_ and _n_ *R*_free_ for neutron refinement were 18.0 and 21.5, respectively, and the final values of _x_ *R*_work_ and _x_ *R*_free_ and _n_ *R*_work_ and _n_ *R*_free_ for XN refinement were 12.5 and 17.0, and 16.2 and 21.2, respectively.

### 3.2. Solving new structures with the generalized XN method   

The generalized XN method has been applied to determine several new structures of biological macromolecules using *CNS* and *PHENIX* and descriptions of these structures and discussions of their scientific significance have been reported elsewhere (Blakeley, Langan *et al.*, 2008 ▶). In this work, two representative new XN structures, photoactive yellow protein (PYP; PDB code 2qws; Fisher *et al.*, 2007 ▶) and diisopropyl fluorophosphatase (DFPase; PDB code 3byc; Blum *et al.*, 2009 ▶), have been chosen for further detailed validation refinements using *CNS* in order to systematically establish the advantages of the XN approach and to expand our set of test structures. PYP and DFPase were chosen because they have very different sizes and data resolutions. The refinements were carried out in the same manner as described above for rubredoxin and the results are represented in Table 2 ▶. The values of *R*_free_ for XN refinement are smaller than or equal to the values for refinement against either X-ray or neutron data alone. An exception is _x_ *R*_free_ for PYP, which is very slightly larger for XN. In addition, the values of Δ_bond_ and Δ_angle_ for XN refinement are also smaller than or equal to their values for refinement against either X-ray or neutron data alone. Improvements in the agreement of the models with the data have therefore not been achieved at the expense of geometry. A third new structure, human aldose reductase (hAR; PDB code 2r24; Blakeley, Ruiz *et al.*, 2008 ▶), was used for further detailed validation refinement with *PHENIX* and confirmed our initial results with *nCNS* as shown in Table 2 ▶. The reason for the apparent arbitrariness in the number of reflections in the test and working sets in Table 2 ▶ is because of the forced matching of reflections in the X-ray and neutron test sets.

**Figure 3:**
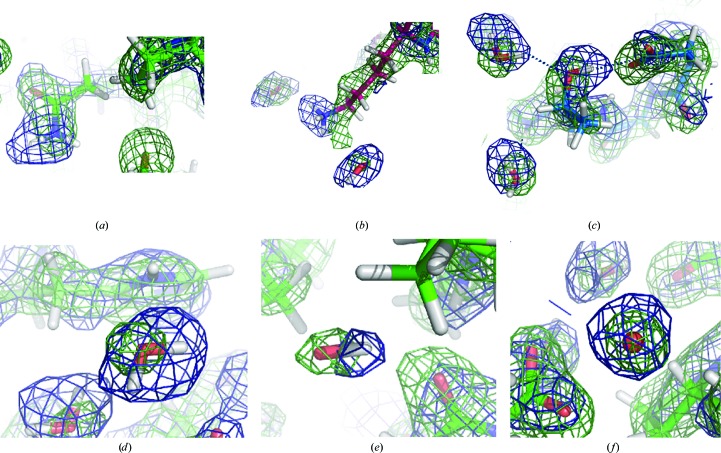
The practical benefits of XN refinement. Selected views of superimposed 2*mF*_o_ − *DF*_c_ neutron scattering (blue) and electron (green) density maps calculated from the XN structure of diisopropyl fluorophosphate (DFPase; resolutions: X-ray, 1.8 Å; neutron, 2.2 Å; contoured at 1.6σ). In (*a*) the N^δ2^ group of the Asn120 side chain scatters neutrons more strongly than X-rays, making it more easily and accurately orientated. In (*b*) note that the terminal amine group of the Lys151 side chain has strong neutron scattering density, whereas the methylene groups of the chain have strong electron density. In (*c*) the orientation of the hydroxyl group of the side chain of Thr102 is clearly indicated by the neutron scattering density. In (*d*)–(*f*) D_2_O molecules appear as either triangles (*d*), ellipsoids (*e*) or spheres (*f*) in the water structure around DFPase. The superposition of the electron-density maps provides complementary information on the location of the O atoms, greatly aiding in the interpretation of these features. In (*d*) the water molecule is ordered and all three atoms are seen. In (*e*) the O—D bond is visible with the second D invisible because of rotational disorder around this bond. In (*f*) the water molecules are completely rotationally disordered.

The benefits of XN refinement and the complementarity of X-ray and neutron data are further illustrated in the superposition of the corresponding neutron and X-ray scattering density maps for DFPase in Fig. 3 ▶. As shown in Fig. 3 ▶(*b*) for a lysine residue and discussed above, the terminal amine group of lysine has strong neutron scattering density, whereas its aliphatic chain has strong X-ray scattering density, leading to a more accurate overall description of the side chain. For asparagine and glutamine residues, because the amino and carbonyl groups of the side chains have similar electron densities, it is difficult to definitively determine their orientation. However, as shown in Fig. 3 ▶(*a*) for an asparagine residue side chain, these groups have very different neutron scattering densities, allowing orientation of the side chain. For threonine and tyrosine residues, because H atoms have little X-ray scattering density, it can be difficult to determine the orientation of the hydroxyl groups of the side chains. However, as shown in Fig. 3 ▶(*c*) for a threonine residue the orientation is clearly indicated by the neutron scattering density.

One of the clearest practical benefits of XN refinement is during water building and refinement. In electron-density maps, owing to the low X-ray scattering power of H atoms, a water molecule is usually represented as a spherical density peak corresponding to the position of the O atom. However, in neutron scattering density maps, D_2_O rather than H_2_O is usually present and owing to the strong scattering contribution from deuterium the associated density may no longer be spherical. During water building and refinement using only neutron data, it is often difficult to interpret these extended neutron scattering density peaks. However, we have found that using both X-ray and neutron data together can greatly help in this interpretation, as demonstrated in Figs. 3 ▶(*d*)–3 ▶(*f*).

## 4. Discussion   

The implementation and testing of a generalized approach to XN refinement, initially in *CNS* and subsequently in *PHENIX*, demonstrates that it provides a more accurate approach to macromolecular structure refinement than refinements using either X-ray or neutron data on their own. In addition to providing more accurate structures, XN refinement has a number of practical advantages, including the easier determination of the orientation of water molecules, hydroxyl groups and some amino-acid side chains. In this approach, we have combined for the first time X-ray, neutron and energy cross-validated maximum-likelihood target functions with gradient-based optimization and simulated annealing methods.

## Figures and Tables

**Table 1:** The values of _x_ *R*_free_ and _n_ *R*_free_ at the start and end of refinement of the published model of the W3Y rubredoxin mutant after different levels of distortion have been applied The refinements were carried out with *nCNS* using either X-ray or neutron data alone or both X-ray and neutron data. A distortion level of 0 corresponds to no distortion and a level of 9 corresponds to a mean position shift of 1.05, a mean ADP shift of 8 and a mean occupancy shift for labile H atoms of 0.5. The values of _x_ *R*_work_ and _n_ *R*_work_ have been omitted for clarity.

			X-ray refinement		Neutron refinement		XN refinement	
Distortion level	Start _x_ *R*_free_	Start _n_ *R*_free_	End _x_ *R*_free_	End _n_ *R*_free_	End _x_ *R*_free_	End _n_ *R*_free_	End _x_ *R*_free_	End _n_ *R*_free_
0	0.226	0.251	0.217	0.285	0.250	0.258	0.212	0.246
1	0.245	0.269	0.217	0.281	0.248	0.256	0.211	0.248
2	0.309	0.314	0.218	0.284	0.249	0.257	0.211	0.247
3	0.380	0.359	0.218	0.284	0.248	0.256	0.210	0.245
4	0.432	0.400	0.219	0.285	0.251	0.257	0.210	0.245
5	0.470	0.435	0.227	0.287	0.252	0.258	0.220	0.248
6	0.501	0.463	0.219	0.284	0.247	0.251	0.211	0.246
7	0.526	0.489	0.255	0.293	0.266	0.260	0.225	0.250
8	0.543	0.511	0.276	0.314	0.288	0.292	0.240	0.263
9	0.559	0.529	0.258	0.299	0.269	0.270	0.229	0.256

**Table 2:** Statistics at the end of refinement of DFPase and PYP using *nCNS* and of hAR using *PHENIX* The refinements were carried out using either X-ray or neutron data alone or both X-ray and neutron data. All data were collected at room temperature.

	X-ray	Neutron	XN X-ray only	XN neutron only
*R*_work_/*R*_free_				
DFPase	0.234/0.260	0.278/0.348	0.233/0.252	0.264/0.315
PYP	0.206/0.228	0.253/0.285	0.215/0.230	0.262/0.277
hAR	0.135/0.162	0.263/0.306	0.131/0.165	0.259/0.298
_bond_ ()				
DFPase	0.005	0.004	0.005	
PYP	0.006	0.005	0.005	
hAR	0.008	0.001	0.011	
_angle_ ()				
DFPase	1.02	1.02	1.01	
PYP	0.98	0.99	0.99	
hAR	1.33	0.68	1.21	
No. of reflections in working sets				
DFPase	23412	11478	34890	
PYP	37127	2519	39646	
hAR	31524	11883	43407	
No. of reflections in test sets (% of total)				
DFPase	1237 (4.4)	564 (3.4)	1801	
PYP	1943 (4.6)	125 (3.4)	2068	
hAR	2952 (9.4)	992 (8.4)	3944	
Completeness (last shell) (%)				
DFPase	92 (74)	82 (73)		
PYP	97 (82)	89 (79)		
hAR	98 (86)	74 (58)		
Resolution range (last shell) ()				
DFPase	20.01.85 (1.941.85)	20.02.2 (2.32.2)		
PYP	20.01.1 (1.141.10)	30.02.5 (2.642.50)		
hAR	33.61.75 (1.811.75)	40.12.2 (2.322.20)		
